# A nonendemic analysis of the patterns and prognosis of de novo metastatic nasopharyngeal carcinomas in older patients aged ≥ 65 years

**DOI:** 10.1038/s41598-022-12368-1

**Published:** 2022-05-18

**Authors:** Baoqiu Liu, Mingxing Zhang, Yanqing Cao, Zhe Wang, Xicheng Wang

**Affiliations:** Department of Oncology, The First Affiliated Hospital of Guangdong Pharmaceutical University, Guangzhou, 510062 China

**Keywords:** Cancer, Oncology

## Abstract

This study aimed to investigate the prognostic factors related to overall survival (OS) and cancer-specific survival (CSS) in patients with de novo metastatic nasopharyngeal carcinoma (NPC) aged ≥ 65 years in nonendemic areas. The Surveillance, Epidemiology, and End Results database was queried for elderly patients with M1 stage NPC at initial diagnosis between 2004 and 2016. This study examined 100 patients and evaluated the relationship of sex, age, race, pathological grade, T stage, N stage, sequence number, site of metastasis, number of metastatic organs, and other related factors with OS and CSS. The median survival and follow-up time were 10 and 48 months, respectively. The survival curves for race, bone metastasis, radiation, and chemotherapy significantly affected OS on the log-rank test. Advanced N stage and liver metastasis may be associated with poor survival. Race, bone metastasis, and chemotherapy were independent prognostic factors of OS. Bone metastasis was associated with poor survival. The survival curves for CSS were significantly different between races, N stage, sequence number, and bone metastasis. In Cox regression multivariate analysis, only sequence number had an independent effect on prognosis. This study revealed that chemotherapy prolonged survival in elderly patients with metastatic NPC, whereas bone metastasis shortened survival.

## Introduction

Nasopharyngeal carcinoma (NPC) is a relatively uncommon malignancy. According to the International Agency for Research on Cancer, there were approximately 129,000 new cases of NPC in 2018, accounting for only 0.7% of all cancers diagnosed that year^1,2^. NPC has a unique regional distribution, and its endemic regions are mainly in Southeast and East Asia, especially Southeast China, where the incidence of NPC ranges from 15 to 50 cases per 100,000 annually^3^. It is worth noting that nonendemic areas, such as the United States and Europe, have low incidences. Nevertheless, according to the 2020 Surveillance, Epidemiology, and End Results (SEER) Cancer Statistics, the incidence of oral and pharyngeal tumours has increased in both men and women (1.2% and 0.5%, respectively)^4^. A previous study reported that distant NPC metastasis occurred in approximately 15% of patients at initial diagnosis^5^.

With advances in therapeutic strategies and developments in radiation technology, remarkable results have been achieved in the treatment of NPC; however, there is not much information on NPC in elderly patients. Chunlin et al. stated in a retrospective study that the clinical stages of enrolled elderly patients with NPC comprised stages II–IVB but not stage IVC^6^, showing that concurrent chemoradiotherapy alone was the gold standard for treatment. Disappointingly, however, the 5-year OS, PFS, LRFFS, and DFS did not improve in the trial results from the same authors^6^. Nevertheless, Shyh-An Yeh described the general conditions of patients with stage IVC metastases but did not specifically study elderly patients^7^. In contrast to Wang's findings, Shyh-An Yeh implied that radiotherapy was a treatment worthy of consideration and that radiotherapy combined with chemotherapy may yield a survival benefit^7^. However, none of the aforementioned clinical trials delicately studied elderly patients with distant NPC. The main reason why there are few studies on elderly patients with NPC is that they have poor tolerance to treatment, various complications, and high treatment risks. Therefore, there is an urgent need to understand the basic disease profile of NPC and the role of radiotherapy and chemotherapy in patients with nonendemic advanced NPC aged ≥ 65 years. To fill the current gaps in knowledge regarding this matter, this study aimed to collect and statistically analyse a small sample of such patients from a real-world study.

## Materials and methods

### Ethics statement

Informed patient consent was not required for the use of our data from the SEER database. Our study was approved by the Ethical Committee and Institutional Review Board of the First Affiliated Hospital of Guangdong Pharmaceutical University. All methods were performed in accordance with relevant guidelines and regulations.

### Databases and study population

This retrospective study selected patients from an NPC database, which was derived from the Surveillance, Epidemiology, and End Results (SEER) database. This database provides information on cancer statistics in populations from the United States. The SEER 18 database was obtained in the SEER program using SEER*Stat software version 8.3.8 (www.seer.cancer.gov/seerstat). The SEER database was queried for patients with NPC between 2004 and 2016. The inclusion criteria were (1) an age of ≥ 65 years; (2) pathologically confirmed NPC; and (3) distant metastasis (M1 stage) as confirmed by the 6th or 7th edition of the American Joint Committee on Cancer (AJCC) staging system. The exclusion criteria were (1) death from unknown causes; (2) a confirmed autopsy; and (3) a diagnosis by the Registry of Deaths.

Demographic, diagnostic, and therapeutic information was obtained from the SEER database using variables comprising ‘sex’, ‘age at first diagnosis’, ‘histologic type’, ‘race’, ‘AJCC 6th stage’, ‘marital status’, ‘chemotherapy record’, ‘radiation record’, ‘sequence number’ and 9 other variables. The sequence number was the frequency of primary malignant tumours. The presence of one primary tumour was defined as nasopharyngeal carcinoma. Others were defined as having two or more malignancies, including nasopharyngeal cancer. A detailed comparison of stages between the 6th and 7th edition of the AJCC system revealed that only 7 patients had discrepancies in T staging between the systems (the patients who had been classified as T1 staging for the 6th edition were classified as T2 staging for the 7th edition), whereas there were no differences in N staging. To maintain the consistency of T and N staging results, the staging was unified according to the staging of the 6th edition of the AJCC, which did not affect the final treatment plan. Based on the World Health Organization (WHO) pathological staging system, histology was divided into keratinizing squamous cell carcinoma (KSCC, SEER codes 8070, 8071; squamous cell carcinoma), nonkeratinizing carcinoma (NKSCC, SEER codes 8020, 8021, 8072, 8073, and 8082), undifferentiated, anaplastic, large- and small-cell nonkeratinizing carcinomas, lymphoepithelial carcinomas, and basaloid squamous cell carcinomas (BSCC, SEER code 8083).

The primary endpoint was OS, defined as the time from first diagnosis to all-cause death, and the secondary endpoint was CSS, which was defined as the time from first diagnosis to NPC-induced death.

### Statistical analysis

The continuous variable of age was classified into three categories—65–69, 70–74, and ≥ 75 years—and subsequently converted into these categorical variables. Moreover, the number of organ metastases was defined as a new variable derived from metastasis to the four following organs: bone, lung, liver, and brain. All categorical variables, excluding OS and CSS, were analysed using Student’s t test or variable analysis. The Kaplan–Meier method was used to draw survival curves, and the log-rank test was used to compare the survival differences of different variables. Independent prognostic indicators of OS and CSS were used in a multivariable Cox regression analysis. SPSS version 16.0 was used for statistical analysis. The Kaplan–Meier curves were described by R 4.1.2. A double-sided *p* value < 0.05 was considered statistically significant.

## Results and discussion

### Patient characteristics

According to the abovementioned inclusion and exclusion criteria, 100 patients (82 men [82%]; median age, 73 [range, 65–88] years) with metastatic NPC were selected from the SEER database. NPC was responsible for 54% of mortalities in this study. Most patients in this study were white (53%), followed by Asian, Pacific Islander, American Indian, and Alaska-native (35%), with black people being the least common (12%). Poorly differentiated NPC was the most prevalent pathological NPC subtype (33%), and T4 and N1 stages were the most prevalent TNM stages, noted for 32 (32%) and 31 (31%) patients, respectively. Advanced T stage (T3–4) was more common than early T stage (T0–2) (52% vs. 30%), and late N stage (N2–3) was less common than early N stage (N0–1) (38% vs. 57%). Among the 100 patients screened, 2 lacked specifics on the distant metastatic sites. The most common metastatic organs were the lungs (46, 46%) and bones (41, 41%). Lymph node (23, 23%) and liver (29, 29%) metastases were not as common as lung and bone metastases, and brain (7, 7%) metastases were the least frequent (Table 1). Regarding the distribution of metastasized organs, excluding lymph nodes, 46 (46%) patients had single-organ metastasis, 20 (20%) patients had two-organ metastasis, and 12 (12%) patients had metastasis in three or more organs (Table 2). Moreover, 66 patients received chemotherapy, 52 patients received radiotherapy, and the remaining 25 patients did not receive any treatment. At diagnosis, 43 patients received chemoradiotherapy combined with initial treatment. Table 1 presents the baseline characteristics of all patients.

**Table 1:** Characteristics and univariable analysis of de novo mNPC on OS and CSS in elderly patients (including weighted gender data).

Characteristic	Cases (N, %)	*P*	
		OS	CSS
**Gender**			
Male	82 (82.0)	0.520	0.710
Female	18 (18.0)		
**Weighted gender**			
Male	82 (69.5)	0.409	0.628
Female	36 (30.5)		
**Age**			
65–69	34 (34.0)	0.474	0.526
70–74	25 (25.0)		
75 +	41 (41.0)		
**Race**			
Black	12 (12.0)	0.022	0.200
Asian or Pacific Islander/American Indian/Alaska native	35 (35.0)		
White	53 (53.0)		
**Marital status**			
Widowed/divorced/ separated single	27 (27.0)	0.200	0.071
Married	60 (60.0)		
Unknown	13 (13.0)	NA	NA
**Grade**			
I/II	17 (17.0)	0.120	0.313
III	33 (33.0)		
IV	22 (22.0)		
Unknown	28 (28.0)		
**Stage T**			
T0–2	30 (30.0)	0.703	0.563
T3–4	52 (52.0)		
Tx	18 (18.0)	NA	NA
**Stage N**			
N0–1	57 (57.0)	0.313	0.133
N2–3	38 (38.0)		
Nx	5 (5.0)	NA	NA
**Sequence number**			
One primary only	76 (76.0)	0.961	< 0.001
Others	24 (24.0)		
**Metastatic bone**			
None	57 (57.0)	0.017	0.458
Yes	41 (41.0)		
Unknown	2 (2.0)	NA	NA
**Metastatic brain**			
None	89 (89.0)	0.808	0.616
Yes	7 (7.0)		
Unknown	2 (2.0)	NA	NA
**Metastatic liver**			
None	69 (69.0)	0.192	0.480
Yes	29 (29.0)		
Unknown	2 (2.0)	NA	NA
**Metastatic lung**			
None	52 (52.0)	0.115	0.075
Yes	46 (46.0)		
Unknown	2 (2.0)	NA	NA
**Distant LNs**			
Distant LN	23 (23.0)	0.477	0.109
Non distant LN	58 (58.0)		
Unknown	19 (19.0)		
**Radiation**			
None	48 (48.0)	0.541	0.446
Yes	52 (52.0)		
**Chemotherapy**			
None	34 (34.0)	0.291	0.158
Yes	66 (66.0)		

### Survival analyses

Univariate analysis using the t test found that race and bone metastases significantly affected OS (*p* = 0.022 and 0.017, respectively). In contrast, no significant differences were found in age, sex, grade, TN stage, tumour metastatic site, number of organs affected, radiotherapy, or chemotherapy between patients. A univariate analysis of CSS using independent variables revealed that one primary tumour alone significantly affected survival (*p* < 0.001). Considering the large disproportion in sex in this study, we weighted the corresponding data and still did not obtain a significant difference.

Cox regression analyses were used to evaluate the possible predictors of CSS in the patients in this study. These analyses revealed that sequence number was the only independent prognostic factor (hazard ratio [HR]: 0.032, 95% confidence interval [CI]: 0.002–0.683, and *p* = 0.027). Multivariate Cox regression analysis of the aforementioned independent variables revealed that race (HR: 1.445, 95% CI: 1.091–1.912, and *p* = 0.010), bone metastasis (HR: 2.399, 95% CI: 1.369–4.203, and *p* = 0.002), and chemotherapy (HR: 0.325, 95% CI: 0.182–0.581, and *p* < 0.001) were potentially significant independent prognostic factors associated with OS. For de novo metastatic NPC, bone metastasis resulted in a significantly poor prognosis. Table 3 presents detailed data on the aforementioned prognostic factors. Further univariate analyses of the number of metastatic organs did not indicate any statistically significant results (Table 2).

**Table 2:** Characteristics and univariable analysis of the number of metastatic organs on OS and CSS.

Characteristic	Cases (N, %)	*P*	
		OS	CSS
**Number of metastatic organs**			
One	46 (46%)	0.857	0.608
Two	20 (20%)		
Three or more	12 (12%)		
Unknown	22 (22%)

**Table 3:** Multivariable Cox regression analysis of independent prognostic factors for OS and CSS in elderly patients with de novo mNPC.

Endpoint	Variable	*P*	HR	95%CI for HR
OS	Race	0.010	1.445	1.091–1.912
	Bone	0.002	2.399	1.369–4.203
	Chemotherapy	< 0.001	0.325	0.182–0.581
CSS	Sequence number	0.027	0.032	0.002–0.683

**Figure 1:**
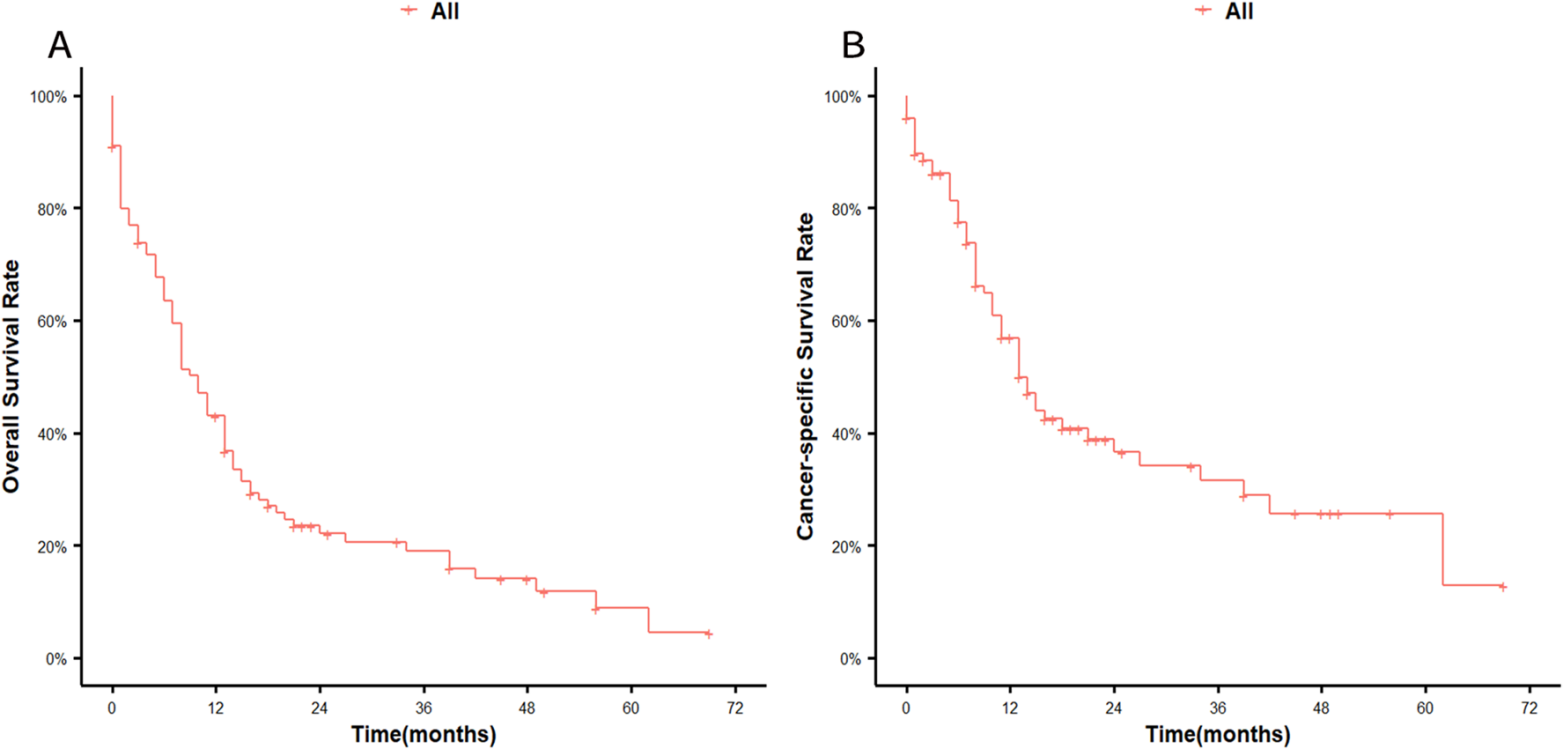
(**A**) Overall survival in elderly NPC, (**B**) cancer-specific survival in elderly NPC.

**Figure 2:**
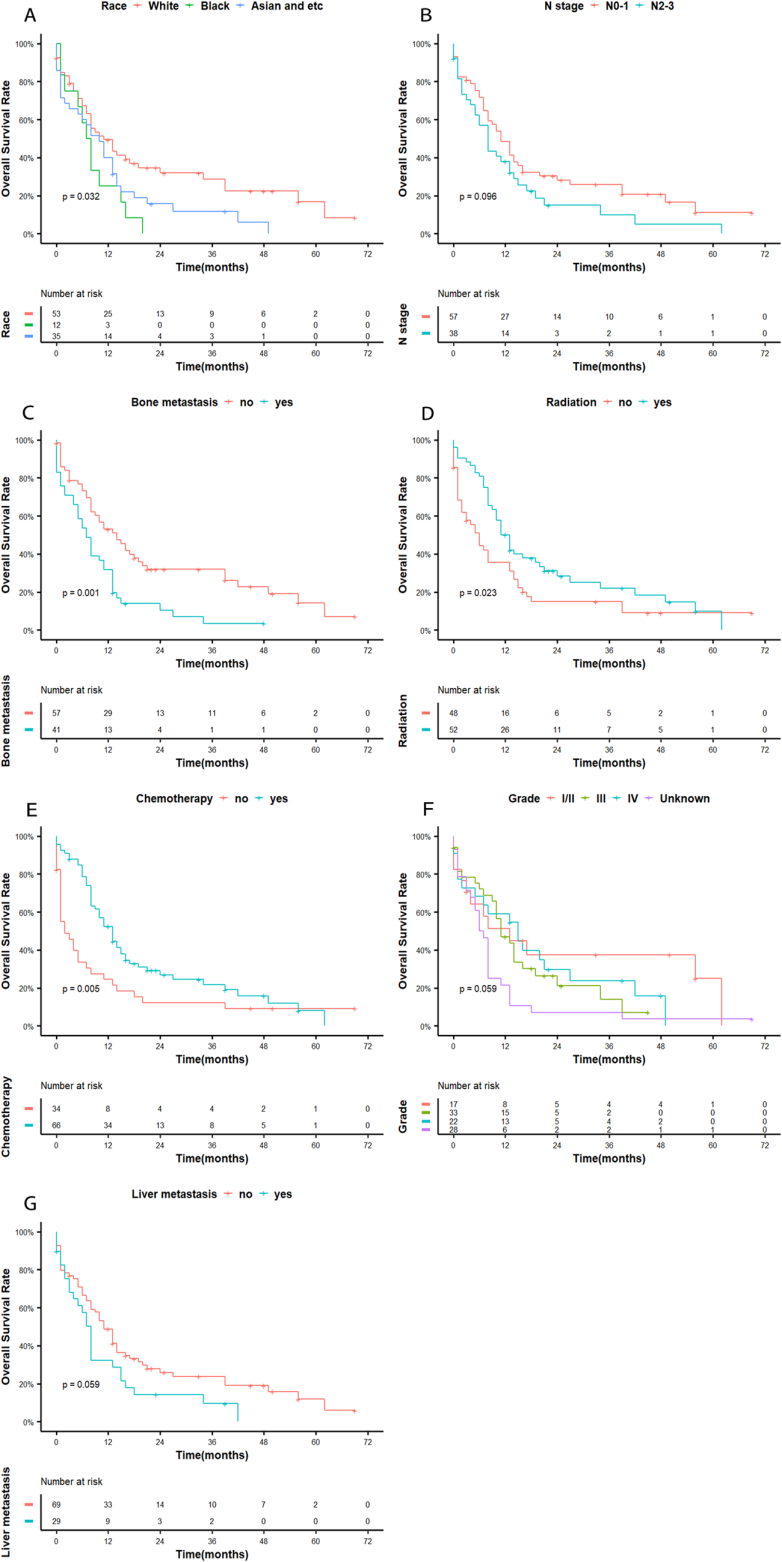
Kaplan–Meier curves showing (**A**) race, (**B**) N stage, (**C**) bone metastasis, (**D**) radiation, (**E**) chemotherapy, (**F**) grade, and (**G**) liver metastasis for OS.

**Figure 3:**
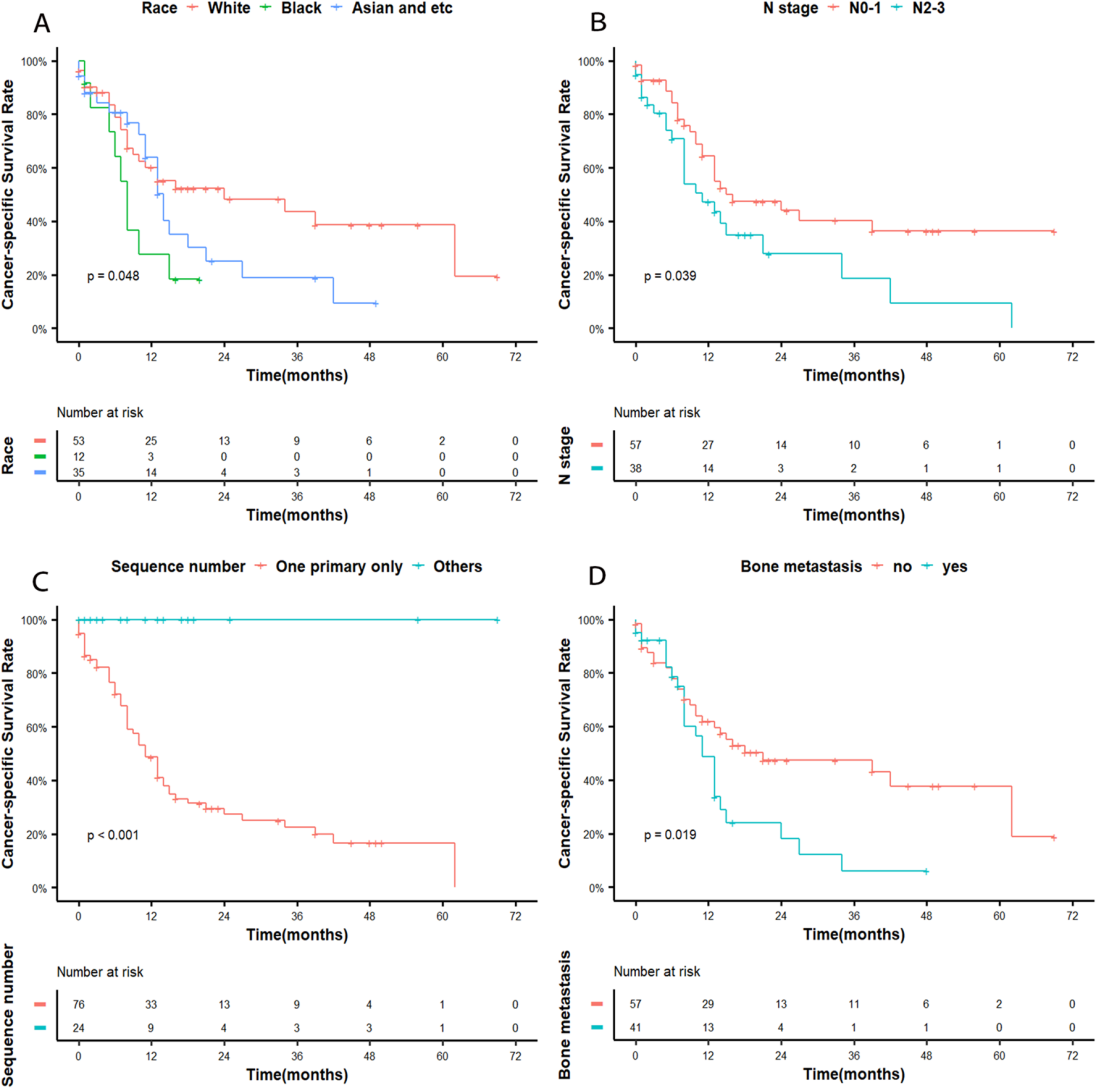
Kaplan–Meier curves are shown. (**A**) Race, (**B**) N stage, (**C**) sequence number, and (**D**) bone metastasis on CSS.

The median survival and follow-up time were 10 and 48 months, respectively. The 12-month, 24-month, and 36-month OS rates were 41%, 15%, and 11%, respectively. Compared with the first year of diagnosis, the second year showed a significant decline in survival (Fig. 1). When we investigated the association between OS and clinical factors using the log-rank test, race (*p* = 0.032), radiation (*p* = 0.023), chemotherapy (*p* = 0.005), and bone metastasis (*p* = 0.001) were significantly associated with survival. Race (*p* = 0.048), sequence number (*p* < 0.001), bone metastasis (*p* = 0.019), and N stage (*p* = 0.039) were significantly associated with CSS in the log-rank test. Although tumour grade and liver metastasis were not shown to significantly affect OS, their P values were approximately 0.05 (both were 0.059) (Fig. 2). N stage may have significance for OS (*p* = 0.096). Figures 2 and 3 illustrate the Kaplan–Meier curves for OS and CSS stratified for each of the possible influencing factors. Survival curves were generated for insignificant factors.

According to the current NCCN and ESMO guidelines, the present standard treatment strategy for stage IVC (TXNXM1) is clinical trials, chemotherapy alone, or a platinum-based combination of chemotherapy with locoregional treatment^8^. However, the current guidelines do not clearly indicate the standard treatment methods for elderly patients with NPC, and few clinical studies have been conducted.

With advancements in medical technology and improvements in the living environment, the lifespan of humans continues to be extended. The age of the population in many countries is increasing, as is the number of NPC cases; therefore, more attention should be given to elderly patients. Some studies have indicated that complications increase with age^9^. Shim et al.^10^ observed that patients aged ≥ 65 years with NPC were more likely to develop renal and ear complications after receiving radiotherapy and chemotherapy than those aged < 65 years. However, several other studies have shown that radiotherapy and chemotherapy do not increase the risk of adverse events^11^. The abovementioned studies are controversial, and more clinical studies and practices are needed to select treatment regimens.

From the statistical data of this retrospective study, it was shown that the incidence of elderly metastatic NPC was higher among males, and the dominant race was white. This result may be caused by regional factors, as the data of this study were collected from the United States Population Oncology Public Database. Ethnicity was an independent prognostic factor. Through Kaplan–Meier curve OS and CSS survival analysis, we found that white people had better survival than Asian, Pacific Islander, American Indian, and Alaska-native people, with black people having the worst survival. This is consistent with the general understanding that the survival effect of the aforementioned factors was greater in Chinese patients with NPC than in white patients with NPC^12–15^. However, due to the small sample size of patients in nonendemic regions, it was difficult to analyse the relationship between race and survival in a more extensive manner.

For histological types, grade III/IV comprised 55%, and grade I/II comprised 17% of the study sample. Grade did not statistically affect survival, but its P value was close to 0.05 [*p* = 0.059], as assessed using a log-rank test for OS, suggesting that survival improved in a large population.

Regarding T stage, the proportions of the T3–4 and N0–1 stages were higher than those of the other T and N stages, respectively. However, there was little difference in the incidence distribution of each stage. T stage and N stage were not shown to be independently correlated with OS and CSS, while early N stage was associated with better survival. The survival curve in this study showed that the survival of patients with N0–1 stage disease was higher than that of patients with N2–3 stage disease in terms of CSS. Simultaneously, curves did not crossover and were separate for OS; therefore, more samples may be needed to better study the survival curves. Unlike previous results, Yali et al. indicated that TN stage did not significantly affect the survival of patients with metastatic NPC in nonendemic areas^16^. Some previous studies have reached a similar conclusion, indicating that N stage may be an independent prognostic factor related to survival^17,18^. Moreover, marital status and grade were not statistically significant factors in this study. Xiong et al. demonstrated that grade and TN stage were insignificant factors in patients with distant metastases, even in areas at high risk of NPC^19^. Two retrospective studies reached completely different conclusions on whether marital status affects survival^20,21^. This characteristic may have different effects on elderly and nonelderly patients.

When analysing the relationship between sequence number and CSS, there was an abnormal result; there was a significant difference between the univariate analysis and the multivariate analysis, and even the Kaplan–Meier curve showed some discrepancies (log-rank test). However, one subgroup of patients was lost to follow-up, affecting the results of this study. Xiaojing et al. implied that multiple primary tumours were a poor prognostic factor, as opposed to single primary tumours^20^. Therefore, we will not discuss the effect of this factor on survival in this study further.

A statistical analysis of multisite metastases showed that bone was the most common metastatic organ, which is consistent with findings from several other studies covering all age groups^22–26^. Because no previous epidemiological statistics for elderly people were found, we were unable to obtain the results of a large sample analysis to compare them with our results. A multivariate analysis showed that bone metastasis significantly affected survival, indicating that bone metastasis is an independent prognostic OS factor, which was inconsistent with the previous result. On the Kaplan–Meier survival curve, bone metastases demonstrated poor OS and CSS outcomes. A paper showed that the incidence of three or more bone metastases was 37.8%, and that of spinal metastasis was 66% at Sun Yat-sen University Cancer Center in China. Three or more bone metastasis sites had poorer survival than fewer metastatic sites (16.2 vs. 32.4 months; HR: 2.52, 95% CI: 1.86–3.42, *p* < 0.001). Spinal metastasis was associated with poorer OS (20.4 vs. 37.9 months; HR: 2.41, 95% CI: 1.72–3.38, *p* < 0.001)^27^. ShaSha He et al. also found that vertebral metastasis was an independent prognostic factor (HR: 1.997, 95% CI: 1.321–3.020, *p* = 0.001) in endemic patients^28^. Based on the abovementioned results, elderly patients with metastatic NPC may have three or more bone metastases or spinal metastases in nonendemic areas. This is probably why the results of our study are different from those of previous population studies. However, Qu Weiling's study obtained opposite results, and meaningful results regarding CSS were obtained using a log-rank test for both liver and metastases^29^. Beyond these sites, other metastatic sites and the number of metastatic organs may significantly affect survival. However, no statistically significant conclusions were obtained using Cox regression multivariate analyses for lymph node, liver, brain, and lung metastases and the number of metastatic organs; this does not mean, though, that these factors have no effect on disease prognosis. A prospective randomized controlled trial found that the number of metastatic lesions and liver metastasis, not the number of metastatic locations, were independent prognostic factors of OS in patients with metastatic NPC^19^. The *p* value of the Kaplan–Meier curve for liver metastasis related to OS was approximately 0.05, and the two survival curves were clearly separated. This result implies that it is possible to obtain meaningful conclusions if the sample size is sufficient. The prognostic value of the number of organs with metastasis (single vs. multiple) is still controversial. The paper reported that single organ metastasis had a better prognosis than multiple organ metastasis^27^. Contrary to this finding, the number of organs with metastasis did not affect the prognosis, but the number of metastatic lesions did, and patients with multiple lesions had a worse prognosis than those with single lesions^16,19,30^. In our study, patients with single organ metastasis did not have better survival, which may be because they may exhibit more metastases within the organ. Jun Ma et al. noted that the size of the lung metastases was an independent prognostic factor for survival in endemic patients^31^. In contrast, Yali reported that liver metastasis had adverse effects on survival, and lung metastasis was not statistically significant on survival^16^. Similarly, lung metastasis may be characterized by small and infrequent lesions. Due to the lack of metastatic information on rare sites and the specific number of metastatic nodules, we were unable to provide more detailed results. These inconsistencies in our study results may be due to the small sample size or differences in epidemiological characteristics between patients, unique biological behaviour in endemic areas, and the specific age range of elderly patients with metastatic NPC.

It is worth noting that in a univariate analysis, there were no significant differences in the effect of chemotherapy on OS, which may be due to the interference of other factors. Chemotherapy at the IVC stage improved OS (HR: 0.40, 95% CI: 0.25–0.65, and *p* < 0.001) but did not significantly affect CSS. Furthermore, chemotherapy was significantly associated with improved OS in this study, which is consistent with other studies, which reported that chemotherapy may improve the OS of older patients with de novo metastatic NPC^16,29^. Unfortunately, radiation was not found to be an independent prognostic factor and only improved OS. Although the results of our study were negative, we cannot completely deny the role of radiotherapy in clinical practice. The survival curves of a previous study showed that radiotherapy resulted in improved OS and CSS in patients with metastatic NPC^7^. Other studies have confirmed that even for de novo metastatic NPC, radiotherapy is still an independent prognostic factor^16,29^. Some studies have demonstrated the efficacy of radical radiotherapy for both the primary tumour and its regional lymph nodes^27,31,32^. According to some indices, such as clinical stage and EBV quantification, for risk stratification, the treatment effect for patients at different levels of risk showed great differences. Jun Ma found that in single metastatic lung tumours from NPC, radiotherapy with or without chemotherapy was superior to chemotherapy alone and worse than surgery with or without chemotherapy on survival (*p* < 0.001)^31^. A study of bone metastatic NPC showed that locoregional radiotherapy improved survival for low- (*p* = 0.006) and intermediate-risk (*p* = 0.005) patients but not for high-risk patients (*p* = 0.918)^32^. Metastasis radiotherapy did not show efficacy on survival. Another study had some findings that patients with stage M1a and M1b could have more survival from chemotherapy plus radical locoregional radiotherapy, not with M1c. No survival benefit was shown with treatment of local metastatic lesions, either with relatively good prognosis for M1a or with the worst prognosis for M1c^19^. In addition, the authors considered that better survival of solitary bone metastasis receiving locoregional radiotherapy may be related to an original better prognosis than radiotherapy^19^. Systemic chemotherapy is the main treatment modality for distant metastatic malignant tumours, while local radiotherapy is aimed at alleviating patients’ symptoms, such as pain and blockage. Therefore, the effect of radiotherapy may have limitations on prolonging survival, and the value of radiotherapy is controversial. It may be that radiotherapy needs to be combined with systemic chemotherapy to benefit nonendemic elderly patients. Three of the abovementioned articles^7,19,33^ were collected from areas in which NPC was prevalent, while two other articles^16,29^ were from nonendemic areas. The median age, age range, and clinical staging of patients in these articles were different from those in our article. This paper presented the different characteristics of elderly patients with metastatic NPC in nonendemic areas and those of patients with nonmetastatic, nonelderly, and/or endemic NPC. Elderly patients are often complicated with a variety of diseases, and clinical workers often only administer conservative treatments for elderly patients with malignant tumours. In this study, we found that chemotherapy can prolong OS, confirming its beneficial role, and that radiotherapy can improve the survival rate and has a certain value for patients.

In previous studies, the effect of age was different for patients aged < 65 years or ≥ 65 years, suggesting that elderly metastatic NPC may have unique characteristics. Our study concluded that young, middle-aged, and elderly patients with NPC have certain similarities and differences, possibly supplementing the current research data on NPC to a certain extent.

Rui et al. reported in his 2020 novel comparative study that in newly diagnosed patients with metastatic NPC, the 2-year OS increased from 54 to 76% after chemoradiotherapy compared to chemotherapy alone^34^. In our study, only 15% of patients survived at 24 months, which is much lower than that reported in the abovementioned study. However, the median age of the two studies differed, 46 years in the previous study versus 73 years in this study. Therapeutic modalities to improve the survival rate and prolong the survival of elderly patients are still required.

This study had some limitations. Due to the limitations of the SEER data obtained, we did not have information on physical scores, EBV-NDA, posttreatment evaluation, posttreatment complications, or tumour recurrence. Therefore, we were unable to conduct further analyses and discussions on the conditions of the patients. The evidence on which we drew our conclusions may be relatively insufficient. Simultaneously, we were unable to clarify the specific chemotherapy regimen and radiotherapy plan and learn more about the specific use of radiation technology; therefore, the present data should be interpreted cautiously. Moreover, we were unable to completely assume that the negative results are necessarily meaningless. We look forward to analysing more data and conducting further prospective studies to provide better guidance for treating metastatic NPC in elderly patients.

## Conclusion

Based on the above statistical analyses and discussion, this study revealed that patients with bone metastases have poorer survival at initial diagnosis than those who do not. Race may influence disease prognosis. Advanced N stage and liver metastasis may reduce the survival rate. Moreover, chemotherapy in patients with NPC extended OS, and survival improved in patients who received radiotherapy. Further studies should be performed to verify the results of this study. The results of our study may be beneficial for making better clinical decisions to improve survival.

## Data Availability

A data availability statement is compulsory for research articles and clinical trials. Here, authors must describe how readers can access the data underlying the findings of the study, giving links to online repositories and providing deposition codes where applicable. For more information on how to compose a data availability statement, including template examples, please visit: https://www.hindawi.com/research.data/#statement.
